# Supplementary material for: Reporting maternal deaths in Anglophone West Africa: A media content analysis of articles published online between 2015 and 2025
Source: PLOS Glob Public Health. 2026 Apr 6;6(4):e0006254. doi: 10.1371/journal.pgph.0006254 (PMC13052848; doi:10.1371/journal.pgph.0006254)
Supplement: S1 File — (DOCX) [file pgph.0006254.s001.docx]

**S1 File** News source selection for ‘Reporting maternal deaths in Anglophone West Africa: A media content analysis of articles published online between 2015 and 2025’

**Supplementary Note S1 File_A**. ChatGPT query and output for online newspapers in Anglophone West Africa

**Query**: “Which newspapers have the highest circulation numbers in Nigeria, Ghana, Gambia, Liberia, and Sierra Leone”?

**Output**:

| **Country** | **Newspapers With Highest Circulation / Reach** |
| --- | --- |
| **Nigeria** | The Punch, The Tide, The Guardian, Complete Sports ([Tribune Online](https://tribuneonlineng.com/top-10-leading-newspapers-in-nigeria-a-comprehensive-overview/?utm_source=chatgpt.com)) |
| **Ghana** | Daily Graphic, Ghanaian Times, Ghanaian Chronicle ([downloads.bbc.co.uk](https://downloads.bbc.co.uk/worldservice/trust/pdf/AMDI/ghana/amdi_ghana7_newspapers.pdf?utm_source=chatgpt.com)) |
| **The Gambia** | The Point, Daily Observer, Today Newspaper ([Wikipedia](https://en.wikipedia.org/wiki/List_of_newspapers_in_the_Gambia?utm_source=chatgpt.com)) |
| **Liberia** | FrontPage Africa (leading investigative daily), Daily Observer (historical), others ([Wikipedia](https://en.wikipedia.org/wiki/FrontPage_Africa?utm_source=chatgpt.com)) |
| **Sierra Leone** | Awoko, Concord Times, The Exclusive ([NationMaster](https://www.nationmaster.com/country-info/stats/Media/Newspapers/List-of-newspapers?utm_source=chatgpt.com)) |

## 🇳🇬 **Nigeria — Highest-Circulation Newspapers**

Nigeria has one of the largest newspaper markets in West Africa, though print circulation has declined in recent years. Major (and widely circulated) national newspapers include:

- **The Punch** — One of Nigeria’s most widely read dailies with substantial circulation and reach.
- **The Tide** — A major daily newspaper with a large regional circulation, especially in southern Nigeria (reported at around 800,000 copies).
- **The Guardian** — Influential national daily (circulation smaller than commercial tabloids but significant in reach and reputation).
- **Complete Sports** — Among the most widely circulated sports-specialist papers, with reported circulation in the hundreds of thousands.

Note: Nigeria’s overall top newspapers vary by region and format (print vs digital), and independent audited print circulation data are limited.

## 🇬🇭 **Ghana — Highest-Circulation Newspapers**

Ghana’s print newspaper market features a mix of state-owned and private titles; among those with higher historic circulation/readership:

- **Ghanaian Times** — One of Ghana’s oldest and most widely distributed state-owned dailies (reported historical circulation around ~80,000).
- **Daily Graphic** — Traditionally the most widely read national newspaper in Ghana (print + extended distribution).
- **Ghanaian Chronicle** — The biggest private newspaper by reported circulation (~45,000).

Note: Like elsewhere, Ghana has seen declines in print sales as digital news grows.

## 🇬🇲 **The Gambia — Leading Newspapers**

Accurate print circulation figures for Gambian newspapers are hard to obtain and often very low due to the small market. However, leading daily titles include:

- **The Point** — One of the main national dailies in The Gambia with wide readership.
- **The Daily Observer** — Historically a leading daily (established in 1992; widely read until operations fluctuated).
- **Today Newspaper** — Another daily in print circulation.
- (Other weeklies/biweeklies include Foroyaa etc., but with much smaller runs.)

Note: Historical estimates suggested Gambian newspaper circulation can be below ~5,000 per day for many titles due to market size.

## 🇱🇷 **Liberia — Leading Newspapers (Smaller Circulation)**

Liberia’s print newspaper market is quite small, and print circulation is limited. Info from existing media profiles shows:

- **FrontPage Africa** — Often described as one of Liberia’s leading newspapers, though reported print circulation has been small (e.g., ~1,500).
- Other important titles (without widely published circulation figures) include **Daily Observer (Liberia)**, **The Analyst**, **New Democrat**, **The Inquirer**, **The Forum**, etc. — historically recognised as leading papers though exact numbers are not published reliably.

Note: Print distribution in Liberia tends to be limited; online presence is increasingly important.

## 🇸🇱 **Sierra Leone — Leading Newspapers**

Accurate audited figures for Sierra Leone are not readily available, but in terms of circulation and visibility, these papers are widely cited as among the most significant:

- **Awoko** — One of the most widely cited newspapers with national reach.
- **Concord Times** — National daily with substantial readership.
- **The Exclusive** — Recognised national paper with regular print editions.

Note: Sierra Leone’s newspaper scene includes many small print titles and limited audited circulation data.

Top of Form

**Supplementary Note_S1 File_B**. ChatGPT query and output for online newspapers in Anglophone West Africa

**Query**: “Which blogs have the highest traffic numbers in Nigeria, Ghana, Gambia, Liberia, and Sierra Leone”?

**Output**:

| **Country** | **Notable High-Traffic/Popular Blogs & Sites** |
| --- | --- |
| **Nigeria** | Linda Ikeji Blog, Naij.com, Nairaland, Vanguard, Punch |
| **Ghana** | GhanaWeb, Pulse Ghana, Yen.com.gh, Modern Ghana, GhanaCelebrities |
| **Gambia** | Seneweb, SeneNews, ThePoint |
| **Liberia** | Geez Liberia, The Bush Chicken, FrontPage Africa, AllAfrica (Liberia) |
| **Sierra Leone** | The Sierra Leone Telegraph, AllAfrica (Sierra Leone) |

Here’s a **regional overview of some of the blogs and online publications with the highest traffic or visibility** in Nigeria, Ghana, The Gambia, Liberia, and Sierra Leone — based on publicly available rankings, blog lists, and web traffic data where possible. Note that in many of these countries formal traffic rankings aren’t regularly published, so some entries are included because they are widely mentioned or recognized as popular in their local markets.

## 🇳🇬 **Nigeria — Most-Visited Blogs / Online Media**

These sites are among the most visited Nigerian blogs or local media platforms (often with blog-like content such as news, entertainment, lifestyle, and community content):

1. **LindaIkejiBlog.com** – One of Nigeria’s most famous entertainment and lifestyle blogs (often ranked very high in local traffic and brand recognition).
2. **Naij.com** – Popular news and entertainment portal with huge monthly visitors (often listed as one of Nigeria’s top sites).
3. **Nairaland.com** – A community forum that behaves like a massive “local blog” with user-generated discussions (very high traffic historically).
4. **Vanguard.NG** – Online arm of a major newspaper that also operates like a high-traffic blog/news site.
5. **ThePunch.ng** – Another high-traffic news site with blog-like content.

In general traffic rankings for all websites in Nigeria are dominated by global platforms like Google and YouTube — but the sites above are consistently among the top **local Nigerian content sources** mentioned in lists of most visited or influential sites.

## 🇬🇭 **Ghana — Popular Blogs and Online Media**

Ghana’s blogging and news ecosystem is anchored by news portals that serve blog-style content:

1. **GhanaWeb.com** – Widely considered Ghana’s most popular online news/blog platform with large traffic volumes.
2. **PulseGhana.com** – Popular entertainment, news, and lifestyle blog.
3. **Yen.com.gh** – Major news and entertainment blog with significant reach.
4. **ModernGhana.com** – Leading local news and blog portal.
5. **GhanaCelebrities.com** – High-profile celebrity gossip blog known in regional rankings.
6. **ZionFelix.net** – Entertainment and lifestyle blog with strong local following.

GhanaWeb regularly tops local website traffic lists among Ghanaian sites — though global platforms like YouTube and Facebook still get the highest overall visits in Ghana.

## 🇬🇲 **The Gambia — Notable Sites (Blogs/News)**

Formal traffic rankings specific to Gambian blogs are limited, but **local news portals and websites with blog-like content** attracting audiences include:

1. **Seneweb.com** – One of the most visited Gambian news sites in recent traffic data (often referenced alongside general web traffic lists).
2. **SeneNews.com** – Another Gambian news portal frequently appearing in local top-visited websites.
3. **ThePoint.gm** – News and commentary site relevant in Gambian media rankings.

Pure travel or personal blogs about The Gambia (e.g., travel diaries) exist, but the sites above are generally the most visible local content hubs resembling blogs/news in The Gambia.

## 🇱🇷 **Liberia — High-Profile Blogs & Online Media**

Liberia’s online space combines traditional news media and blog-style platforms:

1. **Geez Liberia** – A popular Liberian digital media platform covering culture, entertainment, and lifestyle that has received regional recognition and awards.
2. **TheBushChicken.com** – Well-known Liberian news website with wide readership akin to a major blog/news source.
3. **AllAfrica.com — Liberia section** – Aggregated news and blog-style content including Liberia coverage (often cited as top locally relevant site).
4. **FrontPageAfrica.com** – News portal with strong local readership and online presence (often listed in blog lists).

General web rankings in Liberia show global platforms dominating, but the above are among the most recognized **Liberian content sites/blogs** in local media references.

## 🇸🇱 **Sierra Leone — Popular Blogs & Sites**

Traffic data specific to Sierra Leone blogs is sparse, but recognized local blog/news platforms include:

1. **The Sierra Leone Telegraph** – Curated blog-style news source often cited as a top Sierra Leone blog.
2. **AllAfrica.com — Sierra Leone section** – Aggregator covering Sierra Leone news and analysis (often listed among top Sierra Leone online sources).

Beyond these, pure blog traffic data in Sierra Leone isn’t widely published, so aggregated news sites are typically referenced as the **most visible local content platforms**.

**Table S1_File_A**. Number of records and articles retrieved from each country within the five categories.

| **Source** | ***Total per source*** | ***Detailed, obstetric*** | ***Undetailed, obstetric*** | ***Non-obstetric*** | ***Averted maternal obstetric death*** | ***Maternal health as a broad issue*** |
| --- | --- | --- | --- | --- | --- | --- |
| **Nigeria** |  |  |  |  |  |  |
| The Guardian | 8 | 2 | 0 | 1 | 1 | 4 |
| The Punch | 30 | 7 | 2 | 16 | 2 | 1 |
| Daily Trust | 15 | 7 | 0 | 5 | 1 | 2 |
| Vanguard | 20 | 3 | 5 | 13 | 0 | 0 |
| The Nation | 14 | 4 | 0 | 7 | 1 | 2 |
| Premium Times Nigeria | 20 | 3 | 0 | 11 | 0 | 7 |
| Linda Ikeji's Blog | 69 | 7 | 4 | 55 | 2 | 1 |
| Bella Naija | 4 | 3 | 1 | 0 | 0 | 0 |
| International Centre for Investigative Reporting | 1 | 1 | 0 | 0 | 0 | 0 |
| Foundation of Investigative Journalism | 1 | 1 | 0 | 0 | 0 | 0 |
| The Cable | 1 | 0 | 0 | 1 | 0 | 0 |
| Buzzcapital | 1 | 1 | 0 | 0 | 0 | 0 |
| Daily Post | 2 | 2 | 0 | 0 | 0 | 0 |
| Tori.ng | 1 | 1 | 0 | 0 | 0 | 0 |
| The Week | 1 | 0 | 0 | 0 | 0 | 1 |
| Total | 188 | 42 | 12 | 109 | 7 | 18 |
| **Liberia** |  |  |  |  |  |  |
| The Analyst | 0 | 0 | 0 | 0 | 0 | 0 |
| The Inquirer | 2 | 0 | 0 | 2 | 0 | 0 |
| The New Dawn | 2 | 0 | 0 | 0 | 1 | 1 |
| New Public Trust | 1 | 1 | 0 | 0 | 0 | 0 |
| Liberian Observer | 0 | 0 | 0 | 0 | 0 | 0 |
| AllAfrica | 1 | 0 | 0 | 1 | 0 | 0 |
| FrontPageAfrica | 25 | 3 | 2 | 8 | 4 | 8 |
| Total | 30 | 4 | 2 | 11 | 5 | 9 |
| **Sierra Leone** |  |  |  |  |  |  |
| Awoko Times | 0 | 0 | 0 | 0 | 0 | 0 |
| Concord Times | 4 | 0 | 1 | 0 | 0 | 3 |
| Democrat | 0 | 0 | 0 | 0 | 0 | 0 |
| Sierra Leone Telegraph | 5 | 0 | 1 | 1 | 0 | 3 |
| Sierraloaded | 2 | 2 | 0 | 0 | 0 | 0 |
| Cocorioko | 6 | 0 | 0 | 4 | 1 | 2 |
| For di People | 0 | 0 | 0 | 0 | 0 | 0 |
| UNICEF Blog | 1 | 0 | 0 | 0 | 0 | 1 |
| Total | 19 | 2 | 2 | 5 | 1 | 9 |
| **Ghana** |  |  |  |  |  |  |
| Accra Daily Mail | 0 | 0 | 0 | 0 | 0 | 0 |
| Daily Guide | 23 | 3 | 4 | 13 | 0 | 3 |
| Daily Graphic | 0 | 0 | 0 | 0 | 0 | 0 |
| Daily Mail | 8 | 0 | 1 | 6 | 0 | 1 |
| Ghana Web | 3 | 1 | 0 | 0 | 0 | 2 |
| Ghanian Times | 9 | 0 | 2 | 5 | 1 | 1 |
| Graphic Online | 5 | 0 | 0 | 2 | 0 | 3 |
| The Chronicle | 3 | 0 | 0 | 0 | 0 | 3 |
| The Mirror | 2 | 0 | 0 | 2 | 0 | 0 |
| Total | 53 | 4 | 7 | 28 | 1 | 13 |
| **The Gambia** |  |  |  |  |  |  |
| Gambiana | 1 | 1 | 0 | 0 | 0 | 0 |
| The Point | 8 | 1 | 0 | 0 | 0 | 7 |
| What's On Gambia | 1 | 0 | 0 | 0 | 0 | 1 |
| Foroyaa | 5 | 0 | 0 | 0 | 0 | 5 |
| The Voice | 5 | 0 | 0 | 0 | 0 | 5 |
| The Fatu Network | 4 | 0 | 0 | 0 | 0 | 4 |
| The Standard | 15 | 4 | 0 | 0 | 0 | 11 |
| Total | 40 | 6 | 0 | 0 | 0 | 33 |
| **Total** | **330** | **58*** | **23** | **153** | **14** | **82** |

*Note: *Of the 58 articles reporting detailed obstetric events, five were excluded after further screening as content was. In all, 53 articles were included for analysis.*

Bottom of Form
