# Supplementary material for: Reporting maternal deaths in Anglophone West Africa: A media content analysis of articles published online between 2015 and 2025
Source: PLOS Glob Public Health. 2026 Apr 6;6(4):e0006254. doi: 10.1371/journal.pgph.0006254 (PMC13052848; doi:10.1371/journal.pgph.0006254)
Supplement: S2 File — (DOCX) [file pgph.0006254.s002.docx]

**S2 File: Coding Framework and Results of Intercoder Reliability Testing**

**Coding Framework**

| **Question** | **Options** |
| --- | --- |
| Reference number |  |
| Set of similar articles |  |
| Country |  |
| Author name |  |
| Title |  |
| News source |  |
| Hyperlinks |  |
| Delay phase | __ No (if no, exclude paper)  __ Yes (if yes, continue with analyzation)  __ Only Phase I  __ Only Phase II  __ Only Phase III  __ Phase I and II  __ Phase I and III  __ Phase II and III  __ Phase I, II and III |
| 1a. Variety of Sources | _ Family of pregnant woman  __ husband  __ sibling  __ mother  __ mother-in law  __ father  __ friend  __ extended family  __ unspecified  __ Government official  __ Healthcare worker  __ doctor  __ midwife  __ nurse  __ ambulance driver  __ traditional birth attendant  __ other (specify: __________________)  __ Different news source  __ Unaffiliated observer of incident  __ specify: ______________  __ Other |
| 1b. Names sources (barring confidentiality) | Yes, Partly, No |
| 2. Identify (of deceased woman) to ensure verification is possible | Yes, No |
| 3. Respectful reporting/portrayal of death | Yes, Partly, No |
| 4a. Tone | __ Tone  __ neutral  __ persuasive  __ humorous  __ authoritative  __ empathetic  __ Language of reporting  __ vague  __ sensational  __ Anecdotes  __ none  __ relevant to article |
| 4b. Avoids unnecessary details of death | Yes, Partly, No |
| 5. Independent expert consulted | Yes, No |
| 6a. Distinguish between fact and opinion | Yes, No |
| 6b. Journalist does not take a stance | Yes, No |
| 7. Reports firsthand or cites original source | Yes, No |

**Codebook categories for content aligning with the three delays – qualitative analysis (each variable will be either yes/no)**

| Three-delay model-related content included (From main coding frame) | __ Only Phase I  __ Only Phase II  __ Only Phase III  __ Phase I and II  __ Phase I and III  __ Phase II and III  __ Phase I, II and III |
| --- | --- |
| Phase I Delay | __ Socioeconomic and cultural factors  __ lack of birth preparedness  __ lack of trust in healthcare facilities  __ preference for traditional birth customs  __ domestic disputes/violence  __ Status of the woman  __ woman lacked permission to access facility  __ Economic and educational status  __ woman lacked appropriate education  __ woman lacked financial independence  __ low economic status of woman’s family  __ Distance, transport and cost  __ transportation costs a disincentive  __ distance required to traverse a disincentive  __ lack of available transportation  __ hospital fees a disincentive  __ time lost from work a disincentive  __ Quality of care  __ healthcare provider attitudes a disincentive  __ hospital reputation a disincentive  __ lack of comfort at healthcare centre a disincentive  __ long wait times a disincentive  __ Illness characteristics  __ pregnancy complications not considered serious  __ pregnancy complications considered culturally normal  __ previous uneventful birth |
| Phase II Delay | __ Distribution of facilities  __ nearest facility too far  __ facility shortage  __ travel time to nearest facility too long  __ External factors  __ inclement weather  __ road accidents  __ road infrastructure  __ traffic  __ violence  __ police delays  __ Transfer required between facilities  __ delay of transfer caused by inefficient referral system  __ delay of transfer caused by ambulance issues  __Transport method to facility  __lack of safe/any transport options  __transport costs too high |
| Phase III Delay | __ Overall hospital environment delays  __ Care contingent on payment  __ Hospital facility infrastructural issues  __ Inappropriate or poor referrals  __ Long wait times  __ Malpractice  __ Negative attitudes  __ Poor hospital management  __ Ill-equipped facilities  __ shortage of blood  __ shortage of necessary drugs  __ shortage of necessary equipment  __Staffing issues  __ insufficient staff expertise  __ negligence  __shortage of medical staff  __ unavailability of medical staff  __ unspecified |

**Coding Frame: Results of Intercoder Reliability Testing (n=53)**

| **Question** | **% Agreement** | **Cohen’s kappa (κ)** |
| --- | --- | --- |
| Reference number |  |  |
| Set of similar articles |  |  |
| Country |  |  |
| Author name |  |  |
| Title |  |  |
| News source |  |  |
| Hyperlinks |  |  |
| Delay phase |  |  |
| 1a. Variety of Sources | 100.0% | 1.0000 |
| 1b. Names sources (barring confidentiality) | 100.0% | 1.0000 |
| 2. Identify to ensure verification is possible | 100.0% | 1.0000 |
| 3. Respectful reporting/portrayal of death | 94.4% | 0.7798 |
| 4a. Tone | 92.0% | 0.8212 |
| 4b. Avoids unnecessary details of death | 95.6% | 0.7657 |
| 5. Independent expert consulted | 100.0% | 1.0000 |
| 6a. Distinguish between fact and opinion | 100.0% |  |
| 6b. Journalist does not take a stance | 100.0% |  |
| 7. Reports firsthand or cites original source | 100.0% | 1.0000 |
| **Average** |  | **0.9208** |

To calculate Cohen’s kappa (κ) above, we used the formula below:

## Formula

κ = (pₒ − pₑ) / (1 − pₑ)

## Definitions

pₒ (Observed Agreement):
The proportion of times the raters actually agree. It is calculated as the number of agreements divided by the total number of ratings.

pₑ (Expected Agreement):
The proportion of agreement expected by chance alone. It is calculated using the marginal proportions of each rater.

* Kappa could not be computed as application of the formula resulted in a division by 0. However, there was 100% agreement.
