# Supplementary material for: Reporting maternal deaths in Anglophone West Africa: A media content analysis of articles published online between 2015 and 2025
Source: PLOS Glob Public Health. 2026 Apr 6;6(4):e0006254. doi: 10.1371/journal.pgph.0006254 (PMC13052848; doi:10.1371/journal.pgph.0006254)
Supplement: Attachment — Submitted filename: Plos Global Health Review.pdf [file pgph.0006254.s006.pdf]

Thank you for the opportunity to review this important and thoughtful contribution to the field of maternal health and public health reporting. I appreciate the substantial work that has gone into this manuscript and commend the authors for addressing a critical issue in the West African context. It has been a privilege to engage with your research.

Below are my detailed comments:

Introduction

1. Paragraph 4, Lines 35–42

The rationale for selecting Anglophone countries requires strengthening. The manuscript currently states only that "there appears to be no study". The authors should explain how Anglophone media systems differ from Francophone ones, and why these differences are important for understanding maternal health reporting.

2. Paragraph 4, Lines 39–41

The statement that "this analysis will reveal how expectant mothers perceive pregnancy risks" is overstated. Media analysis cannot directly measure women's perceptions. I would strongly recommend reframing this claim to avoid over-interpretation.

Methods

3. Lines 46–48

Although the methods section is generally detailed, some methodological gaps remain. The authors cite Macnamara (2005) but do not describe which components of the Macnamara framework were applied. A stepwise explanation of how this approach informed data extraction, coding, and analysis should be added.

4. News Source Selection

The justification for selecting the specific newspapers and blogs is insufficient. No readership, circulation, or web-traffic data are provided. Please provide objective metrics (e.g., circulation figures, page-view statistics) and explain why other possible sources were excluded.

5. Search Strategy (Lines 86–100)

The search terms used (pregnant woman, maternal mortality) are very narrow and risk missing relevant articles (e.g., expectant mother, woman dies in childbirth). The authors should justify the choice of terms or expand them. Additionally, please clarify why Boolean operators were not employed to enhance the search strategy.

6. Eligibility Criteria (Lines 101–111)

Requiring articles to include the woman's name and contain more than 250 words may introduce selection bias and exclude culturally sensitive reporting. The authors should justify the 250-word threshold with evidence from the literature and discuss the ethical implications of requiring the deceased woman's name.

7. Data Extraction and Reliability

Although the use of Google Sheets and NVivo is appropriate, the manuscript does not describe the number of coders, their training, or intercoder reliability. Furthermore, no reliability statistics (e.g., Cohen's kappa) are reported despite both quantitative and qualitative coding. The authors should report reliability measures or explain how coding disagreements were resolved.

Results

8. The results section is highly descriptive. The authors are encouraged to present the findings more concisely. Consider separating the results into descriptive characteristics and patterns of reporting to improve structure.

9. Qualitative Findings (Lines 225–293)

The qualitative synthesis reads more like a summary of article content than a thematic analysis. The authors should organise the findings around explicit themes (e.g., financial barriers, provider negligence, delays in obtaining care) and present them systematically.

Discussion

10. Paragraph 2, Lines 420–434

The interpretation is generally strong; however, some statements are speculative, such as attributing the rise of blogs to increased pandemic-related readership, without supporting evidence. These claims should be substantiated with references or revised for caution.

11. Paragraphs 3–5, Lines 435–479

Parts of the discussion read as advocacy rather than scientific analysis. To maintain academic rigor, the authors should minimise editorial tone and ensure that interpretations remain firmly grounded in the data.
