# Supplementary material for: Reporting maternal deaths in Anglophone West Africa: A media content analysis of articles published online between 2015 and 2025
Source: PLOS Glob Public Health. 2026 Apr 6;6(4):e0006254. doi: 10.1371/journal.pgph.0006254 (PMC13052848; doi:10.1371/journal.pgph.0006254)
Supplement: Attachment — Submitted filename: Response to Peer review comments_final.docx [file pgph.0006254.s009.docx]

28^th^ January 2026

Prof Catherine Kyobutungi

Prof Manuela de Allegri

Editors-in-Chief

Dr Julia Robinson

Executive Editor

Dr Emmanuel Olamijuwon

Academic Editor

PLOS Global Public Health

Dear Editors,

**Re: Reporting maternal deaths in Anglophone West Africa: A media content analysis of articles published online between 2015 and 2025**

We appreciate the opportunity to revise and resubmit our paper, and the comments provided by the editor and reviewers, which have enabled us to strengthen the manuscript.

We have carefully considered all comments and have addressed each point comprehensively. Below, we provide a point-by-point response to the reviewers' feedback, detailing the revisions made to the manuscript.

We took the opportunity to update the review to include newer articles that have emerged in the media since our first submission, updated the abstract based on new changes made, created new supplementary files, and reviewed language in the entire manuscript to ensure clarity. We also made a change to the title to make it more reflective of the content of the paper.

We believe these revisions have strengthened the methodological rigour, theoretical grounding, and clarity of our study, and we hope the manuscript is now suitable for publication in PLOS Global Public Health.

Thank you for your consideration.

Kind regards,

Aduragbemi Banke-Thomas

(On behalf of all authors)

**Reviewer #1:**

The manuscript addresses an important and underexplored public health issue: media reporting of maternal deaths in Anglophone West Africa. The topic is relevant, timely, and well aligned with the journal’s scope. The study design, a media content analysis, is appropriate for the research question, and the authors present a substantial body of extracted data. However, several methodological gaps reduce the technical robustness of the manuscript in its current form.

***Response****:* Thank you for your detailed and thorough review of our paper. We are thrilled that you agree with us on the relevance and timely nature of our study. We have taken your very helpful comments on board to help us strengthen the paper ensuring that its technical robustness is improved substantially.

Specifically, key components require strengthening to meet publication criteria:

i). The rationale for selecting Anglophone countries is insufficient and needs deeper justification (Introduction, paragraph 4, lines 35–42). The authors should explain how Anglophone media systems differ from Francophone ones, and why these differences are important for understanding maternal health reporting.

***Response****:* Thank you to for the comment. We have revised our rationale for country selection, writing, *“Because language differentially shapes how public health issues are communicated in the media [16,17], any assessment of the media’s potential utility for this purpose must be conducted within a specific linguistic context. With approximately four of five maternal deaths in West Africa occurring in the region’s Anglophone countries [1]…]”* (Page 1, Paragraph 4)*.*

ii). The Macnamara framework is cited but not operationalised, leaving the analytic procedure unclear (Methods, lines 46–48).

***Response****:* We note this comment. Please permit us to flag that Macnamara (2005) is a guidance; not a framework to be used for analysis. As stated in our paper, we closely followed its guidance on media content analysis. See reference: Macnamara J. Media content analysis: Its uses; benefits and best practice methodology. Asia Pacific Public Relations Journal. 2005;6(1):1–34.

iii). News source selection lacks objective justification. Please provide objective metrics (e.g., circulation figures, page-view statistics) and explain why other possible sources were excluded.

**Response**: Thank you for this comment. We have described how circulation numbers were determined by querying ChatGPT and included a supplementary file with its output for reference. The text now reads, *“To select online newspapers, four to five of the leading online newspapers in each country were selected based on their circulation numbers, which can indicate popularity. The circulation numbers were retrieved by querying ChatGPT (OpenAI, San Francisco, California, USA). The output generated was validated by asking colleagues who live in the countries if they agreed that the identified papers were the most widely read in each country. In the case that colleagues local to the country disagree with the generated list, proposition from local colleagues was prioritised”* (Page 3, Paragraph 1)*.*

iv). The search strategy is narrow and may not capture the full spectrum of relevant media reports.

***Response****:* Thank you for this feedback. We had only previously stated the search terms that helped us retrieve the relevant articles. As stated, we pilot tested the search strategy before full deployment and it generated the full spectrum of media reports. This was supplemented by a wide social media campaign to secure additional sources. To address your comment and in the interest of full transparency, we have now detailed all search terms tested and the final ones used to retrieve articles. From our experience in conducting this study, these terms were the most sensitive ones. The text here now reads, *“An initial preliminary search was conducted through an iterative process to pilot test several search terms (“maternal mortality”, “maternal death”, “dead woman”, “pregnant woman”, “pregnancy-related death”, “pregnancy-associated death”, “pregnancy complication” and “obstetric mortality”) and assess relevance of articles retrieved to the study. The preliminary search was conducted using the search tab function of the websites of different news source. Following this, the search terms “pregnant woman”, “maternal mortality”, and “maternal death” were assessed as most sensitive to identify relevant articles, with the presence of one or the other (“pregnant woman” OR “maternal mortality” OR “maternal death”) warranting an assessment of the identified article”* (Page 3, Paragraph 3).

v). Eligibility criteria, especially requiring the woman’s name and a word count threshold, may introduce bias but are not adequately justified.

***Response****:* Thank you for this comment. It is not quite clear how the name could have introduced bias, but we understand your concern with the word count threshold. For the name, we needed this information in the report to ensure that the veracity of the reporting was not in doubt. Simply saying, “a woman” or “Woman X” died could be a fictitious story, especially with blogs. However, a name added lends some credibility to the report. You will see in our PRISMA diagram that we flagged that we excluded one article because we had *“concern on authenticity of report (n = 1)”*. For the word count threshold, as mentioned, we pilot-tested this approach before applying it. Articles <250 words did not have sufficient textual richness to allow us fully to characterise and understand the obstetric cause of death. We have now explicitly stated this as a justification for taking this approach now, writing that *“Our preliminary screening of all retrieved articles revealed that articles with more than 250 words tended to have sufficient textual depth needed for meaningful content analysis, including the identification of themes, frames, and discursive patterns [19]. On the other hand, shorter reports typically lacked the narrative and contextual complexity required for comprehensive understanding of the cause of the maternal death”* (Page 3, Paragraph 4).

To ensure we fully address your concern, we have flagged this as a potential limitation while highlighting the reason for setting the benchmark and stating we do not believe it would have altered our findings. We have done this by adding that “*Fifth, we included articles with 250 words or more. While this approach might have introduced some bias into our findings, our review of the less detailed articles presented in S1 File suggests that, although their inclusion would have increased the number of eligible articles, the overall conclusions would not have changed”* (Page 16, Paragraph 2). You will see in S1 File, we report the number of these undetailed reports (23) including 12 from Nigeria and none from The Gambia.

vii). The qualitative findings read largely as narrative summaries, with insufficient thematic structure.

***Response****:* We have not used thematic analysis but rather framework analysis. We have also used narratology, a defined method used in media content analysis, which involves use of narrative summaries. See reference:

Fludernik M, Pirlet C. Narratology. In: Middeke M, Müller T, Wald C, Zapf H, editors. English and American Studies. Stuttgart: J.B. Metzler, Stuttgart; 2012. p. 225–30. Available from: https://link.springer.com/chapter/10.1007/978-3-476-00406-2_13

vi). Intercoder reliability is not reported, which is essential for both qualitative and quantitative coding.

The manuscript does not include formal statistical analysis. The study relies exclusively on simple descriptive frequencies and qualitative coding. While descriptive statistics are appropriate for a media content analysis, two issues compromise rigour: I). No intercoder agreement statistics (such as Cohen’s kappa or Krippendorff’s alpha) are reported. Given that coders classified articles into delay phases, quality categories, and thematic elements, reliability metrics are essential to ensure coding consistency. Although the analytical approach does not require complex inferential statistics, the absence of reliability metrics and opaque scoring criteria undermines analytical rigour and should be addressed.

***Response****:* We are immensely grateful for your comment. We have now stated clearly that, *“Two coders (KR and AB-T) independently coded all selected articles using the coding sheet. As per best practice, inter-coder reliability for the quantitative content analysis was assessed for agreement between coders using Cohen's kappa (κ) [19]. Following an iterative process, we achieved an inter-coder reliability (κ) of 0.92 [S2 File], with κ >0.75 accepted as excellent [28]*” (Page 5, Paragraph 1). We have also included a supplementary file detailing our calculation of κ. Please note that for the qualitative component, we deployed a mostly inductive coding. While intercoder reliability ensures multiple coders agree on data interpretation, it is generally considered unnecessary or inappropriate in purely interpretive or inductive studies that embrace subjective, multiple realities. Researchers argue that calculating numerical reliability (e.g., Cohen's Kappa) imposes quantitative, positivist, or *"single truth"* standards on inherently subjective, interpretive data. In lieu, we implemented a detailed audit trails and debriefing meetings. This ensured that we were able to represent the multiple realities of the text from the lens of the two coders. It is their blended understanding of what has been reported in the media that has been presented as findings. We have explained this in the manuscript and included relevant references to justify our approach.

The authors provide detailed supporting files (S1–S3), which include: The full list of included articles and extracted variables, coding outcomes, reporting-standard scoring, assessment of language and sensationalism, and Identification of duplicate stories. These files are comprehensive and align with PLOS’s open-data requirements. However, a formal codebook defining the coding categories is not provided, and the absence of intercoder reliability makes it unclear how consistently the coding was applied. While the data themselves are available, the transparency of the coding framework could be improved to enable full reproducibility.

***Response****:* Thank you for this comment. We have included the codebook used for analysis [S2 File].

The manuscript is generally written in clear English. The structure follows a conventional academic presentation, and the argument is easy to follow. However:

i). Some sections, particularly the Results, are overly descriptive and would benefit from clearer organisation into descriptive characteristics and patterns of reporting.

ii). The qualitative results require more thematic clarity.

iii). The Discussion section occasionally adopts an advocacy tone and includes speculative statements that should be supported with citations or reframed cautiously.

The conclusions are generally consistent with the descriptive findings presented, but some claims, particularly in the Discussion, are speculative and require either stronger evidence or more cautious framing. With these issues addressed, the manuscript could provide a valuable contribution; however, substantial revision is required for methodological and interpretive rigour.

***Response****:* Thank you very much for your very helpful comment which has helped us further strengthen our paper. We have reviewed the results and discussion sections. Please note that the results are organised in line with the objective, starting with an overview, then moving what about maternal mortality is being reported before going to how it is being reported. The results should be descriptive. Please see the published journal guidelines (<https://journals.plos.org/globalpublichealth/s/submission-guidelines>). As we used framework analysis, it is certainly not unusual to use codes, as opposed themes to present findings. This was not a thematic analysis. It is also not unusual for a discussion section in an academic paper to adopt an advocacy tone, especially in the implication for policy and practice. This is more so relevant in a paper on media reporting. The main caveat is that such advocacy must be evidence-based, balanced, and closely tied to the study’s findings. As such and linked to your comment, we have reviewed the entire discussion to ensure that speculative statements not backed by citations have either been removed or had a citation added to them (Page 13 -18).

**Reviewer #2**

Summary:

This manuscript addresses an important topic of how maternal deaths are reported in Anglophone West Africa and what this reveals for maternal mortality surveillance and accountability. The study is timely, theoretically grounded, and methodologically innovative in linking media analysis with maternal health frameworks. However, the manuscript would benefit from clearer justification of methodological choices, a balanced presentation of results, stronger critical reflection on background and discussion, and more actionable implications for both public health and media practice.

***Response****:* Thank you for your thoughtful comment. We are pleased that you see that the manuscript addressed an important topic and is timely, theoretically grounded, and methodologically innovative. We have taken on board your very helpful comments to help us strengthen the paper providing clearer justification of methodological choices, a balanced presentation of results, stronger critical reflection on background and discussion, and as deemed relevant, additional actionable implications for public health and the media.

Introduction:

“Another source of information on various kinds of health-related topics, including maternal deaths, is the Media.” The term “media” is broad and requires further elaboration. The authors should clearly define what types of media are included (e.g., newspapers, blogs, social media, investigative journalism) and briefly explain how media functions as a source of maternal health information.

***Response****:* Thank you for this comment. We agree that the term media is broad. We have now broadly categorised it and highlighted the value of digital/new media for public health, making it clear subsequently that digital/new media is the type included in our analysis while briefly explaining how media functions as a source of maternal health information. We have written: “*The media, encompassing both traditional (e.g. newspaper, television, and radio) and new/digital media (e.g. online newspaper, blogs, etc.), is another source of information on health topics, including maternal deaths [10,11]. The media plays a crucial role in shaping the public’s understanding and perception of public health issues, with digital media, deemed to be particularly effective in this regard [12–14]. Beyond informing the public, the media exerts significant influence on real-world events by holding power to account, amplifying marginalised voices, and guiding the decisions of policymakers. In the area of maternal health, the media has been used to convey the severity of the maternal mortality crisis, impact of implemented interventions, and the need for improved health policies and legislation [15]. Unlike data collected via MPDSR, those gathered by the media do not come at a cost to the government*”*.* (Page 1, Paragraph 3)

“Newspapers, social media, and blogs play a crucial role in how the public understand and perceive public health issues.” Social media is a heterogeneous category. The authors should specify which platforms are included under “social media” and clarify how these platforms differ from newspapers and blogs in shaping public health perceptions.

***Response****:* We have removed social media completely to avoid distracting readers from the focus of the paper, since we did not review social media in the paper (Page 1, Paragraph 3).

“Both methods help improve understanding of the causes of maternal deaths in sub-Saharan Africa, however, implementation has been fraught with numerous challenges including cost associated with routine data collection, blame culture, and so on.” This statement would benefit from further elaboration. The authors should expand on the key challenges associated with verbal and social autopsy methods (e.g., sustainability, data completeness, blame culture, feasibility in low-resource settings) to better justify the need for alternative data sources such as media reports.

***Response****:* Thank you for this comment. We have added expanded this sentence now to strengthen the justification, writing, “*Both methods help improve understanding of the causes of maternal deaths, however, its implementation in many West African countries has been fraught with numerous challenges including cost associated with routine data collection, which makes it difficult for governments to sustain [7–9]*” (Page 1, Paragraph 2)*.*

“While a previous article by Gugsa et al. (2015) examined newspaper coverage of maternal health more generally…” The manuscript would benefit from a clearer articulation of its novelty. The authors should explicitly state how this study differs from and advances prior media-based research, particularly in terms of innovation, geographic focus, or analytical framework.

***Response****:* Thank you for this very important comment. There is no previous research that has conducted a media content analysis in Africa. We have clearly stated, *“Given the high burden of maternal mortality and the persistent challenges with implementing community-based MPDSR in West Africa [1,9], it is important to explore the potential of the media as an alternative data source for understanding causes of maternal deaths in the region. To the best of our knowledge, no study reviewing media content on maternal deaths has been published to date”* (Page 1, Paragraph 4)*.*

“Our objective in this study was to conduct a media content analysis to understand what about and how maternal deaths are reported across print and electronic media outlets in Anglophone West Africa.” What are print and electronic media outlets, what is the importance of these media outlets on maternal health, and how did authors come to have print and electronic media outlets? Authors need to explain this.

***Response****:* Thank you for picking this comment. We have rephrased the objective to make sure it clearly elucidates the focus of our paper. We are clear from page 1, paragraph 3 that digital media has higher value in public health. The objective now reads, “…*our study aimed to conduct a media content analysis to examine what about and how maternal deaths are being reported in digital media in Anglophone West Africa”* (Page 1, Paragraph 4)*.*

Method:

“Various individual factors relating to delay in seeking care ….contribute to maternal deaths in the sub-region .” This statement appears to rely on limited citation. The authors should draw on a broader body of literature to support claims on the information of individual factors related to delay in seeking care.

***Response****:* Thank you for this comment. This statement is already drawing from a large recent scoping review which captures the entirety of the statement. To ensure this is not missed on the reader, we have now specified, “*According to 2022 recent scoping review, various individual factors relating to delay in seeking care, health facility relating to sub-optimal triage, monitoring, and referral, and wider health system factors such as transportation between health facilities contribute to maternal deaths in the sub-region [20]”* (Page 2, Paragraph 4)*.*

“First, in each country, four to five of the country’s leading online newspapers were selected based on circulation numbers, which can indicate popularity.” The selection criteria for “leading” newspapers require further clarification. The authors should explain how circulation or popularity was determined and provide supporting evidence or references.

**Response**: Thank you for this comment. We have described how circulation numbers were determined and included a supplementary file with the output for reference. It now reads, *“To select online newspapers, four to five of the leading online newspapers in each country were selected based on their circulation numbers, which can indicate popularity. The circulation numbers were retrieved by querying ChatGPT (OpenAI, San Francisco, California, USA). The output generated was validated by asking colleagues who live in the countries if they agreed that the identified papers were the most widely read in each country. In the case that colleagues local to the country disagree with the generated list, proposition from local colleagues was prioritised”* (Page 3, Paragraph 1)*.* We have expanded content in S1 File to provide more details on the new source selection process and output.

“After this campaign, additional sources including additional blogging sites and investigative reporting sites were identified.” The inclusion of blogs and investigative reporting sites alongside newspapers requires clearer justification. The authors should explain how these sources align with the study’s objectives and analytical framework.

***Response****:* Online newspapers and blogs are both digital media, which we have now clearly stated as the source of focus for this study and stated it in the objective.

“An initial preliminary search was conducted to test search terms and assess articles retrieved. For each news source, the search terms “pregnant woman” and “maternal mortality” were used within the search tab function of the website.” The authors should justify the selection of these search terms and clarify whether other relevant terms (e.g., maternal death and postpartum period) were considered.

***Response****:* As mentioned, we trialled several terms before settling for “*pregnant woman”*, *“maternal death”* and *“maternal mortality”*. AS this comment was raised by multiple reviewers, we have now detailed all search terms considered and the final ones used to retrieve articles. From our experience in conducting this study, these terms were the most sensitive ones. The text here now reads, *“An initial preliminary search was conducted through an iterative process to pilot test several search terms (“maternal mortality”, “maternal death”, “dead woman”, “pregnant woman”, “pregnancy-related death”, “pregnancy-associated death”, “pregnancy complication” and “obstetric mortality”) and assess relevance of articles retrieved to the study. The preliminary search was conducted using the search tab function of the websites of different news source. Following this, the search terms “pregnant woman”, “maternal mortality”, and “maternal death” were assessed as most sensitive to identify relevant articles, with the presence of one or the other (“pregnant woman” OR “maternal mortality” OR “maternal death”) warranting an assessment of the identified article”* (Page 3, Paragraph 3).

“Articles with more than 250 words tended to have sufficient detail needed to help us realise the objective of our analysis.” A stronger justification is needed for the 250-word cutoff. The authors should indicate whether this decision was informed by pilot testing or prior literature and acknowledge potential exclusion of shorter but influential reports.

***Response****:* Thank you for this important comment. We had previously made it clear that the benchmark was established following a pilot test. We have now expressly stated this and strengthened our justification, writing, *“Our preliminary screening of all retrieved articles revealed that articles with more than 250 words tended to have sufficient textual depth needed for meaningful content analysis, including the identification of themes, frames, and discursive patterns [19]. On the other hand, shorter reports typically lacked the narrative and contextual complexity required for comprehensive understanding of the cause of the maternal death”* (Page 3, Paragraph 4).

“A detailed report of maternal death due to obstetric causes…”. The manuscript should clearly define obstetric versus non-obstetric causes of maternal death, with brief examples, to improve clarity for readers.

***Response****:* Thank you for this very helpful comment. We have expanded the narrative here and provided examples. The statement now reads, *“(1) a detailed report of maternal death due to obstetric causes, (e.g. bleeding and high blood pressure, as defined by the WHO [1])*” (Page 3, Paragraph 4). We also defined and provided examples for what we now refer to as “accidental/incidental causes” as opposed to “non-obstetric causes” (e.g. road traffic accident, murder) (Page 3, Paragraph 4).

Results:

“Of all 46 included articles, there were 29 (63%) online newspaper articles [26 29,31,32,34,37,38,40–43,46,49,50,53–63,69,70], 15 (33%) blogs [25,30,33,36,44,45,47,48,51,52,64–68], and two (4%)investigative reports [35,39].” Given the dominance of newspaper articles and the limited number of investigative reports, the authors should acknowledge this imbalance and discuss its implications for interpretation.

***Response****:* Thank you for this comment. We agree that this difference should be interpreted and its implication for practice discussed, but do not agree with your characterisation of this as an “imbalance”. As we see it, it is simply a finding that more of the deaths are being reported in online newspapers. Please see discussion, where we have expanded text to read *“While online newspapers constitute the predominant source of detailed reports on maternal deaths in Anglophone West Africa, blogs have increasingly emerged as a formidable platform for maternal death narratives, especially since the COVID-19 pandemic, with examples retrieved from Liberia, Nigeria, and Sierra Leone”* (Page 14, Paragraph 1). We have also added that *“For online investigative reports, we only retrieved two [41,45]. We reckon that this might be a reflection of the challenges faced with investigative journalism across many parts of SSA, including lack of funding, government influence, media censorship, insecurity, threats and lawsuits, and poor access to information [86–88]”* (Page 14, Paragraph 1).

“Across all articles, Nigeria had 31 unique maternal deaths published across 35 articles on maternal death (76%) [27,29,31–37,39–47,49,51–55,57–59,61–63,66–70], followed by The Gambia with three unique stories across four articles (9%) [25,28,56,60], Liberia with three unique stories in three articles (7%) [26,30,48], Ghana with two unique stories in two articles (4%) [38,50], and Sierra Leonne also with two unique stories in two articles (4%) [64,65]”. As the dataset is heavily weighted toward Nigeria, the authors should avoid strong cross-country comparisons and clearly state that findings primarily reflect Nigerian media coverage.

***Response****:* Thank you for this comment. We agree that despite us setting out identify articles across all Anglophone West African countries, the dataset is heavily weighted towards Nigeria. This reflects the field. We also understand your caution on strong cross-country comparison, and as such we have checked to make sure we avoided any such comparison while restructuring the results in a way that makes it easier for readers to appreciate observed patterns in individual countries that have been included in our study. Please look at the updated results.

“Of the 46 articles included, there were 10 sets of articles reporting the same maternal death.” The authors should discuss whether repeated reporting of the same deaths may have limited the diversity of narratives and consider whether the search strategy could be expanded to capture greater variation.

***Response****:* Thank you for this extremely important point. We have detailed how we approached duplicates in our study, writing in the Methods section that, *“In instances where a specific maternal death was reported by more than one news sources, we analysed content on ‘what about’ the maternal death a unique case, while citing the multiple sources together. However, we analysed ‘how’ the maternal death as individual reports, citing the multiple sources separately. We chose this diverging approach since the case being reported is one but how the same case is being reported may vary”* (Page 5, Paragraph 2).

Discussion:

“More than 30% of the world’s maternal deaths occur in this region.” This statement should be supported with additional or more recent references to strengthen its credibility.

***Response****:* The most recent reference from the WHO is already cited. See reference: WHO, UNICEF, UNFPA, World Bank Group, UNDESA/Population Division. Trends in maternal mortality 2000 to 2023: Estimates by WHO, UNICEF, UNFPA, World Bank Group and UNDESA/Population Division. New York; 2025. Available from: <https://data.unicef.org/wp-content/uploads/2025/04/Trends-in-maternal-mortality-2020-2023.pdf>

“As per findings of our analysis, majority of the articles identified were published in Nigeria. This is not entirely surprising, as the annual number of maternal deaths occurring in Nigeria (75,000 in 2023) is significantly higher than estimates from the other Anglophone countries in West Africa (Cumulative of 4,410 in 2023) [1].” This is not a strong justification. The authors need to provide a better rationale for this.

***Response****:* Thank you for your comment. This is not a justification or rationale. It is a potential explanation of the over-representation of Nigeria media reports. It could be deemed logical that if you have substantially more deaths in a country then that may explain why there is more reporting. We wrote that, *“Severity of a country’s maternal mortality may influence how the media in that country reports the public health issue”* (Page 14, Paragraph 1).

“In our study, more than 80% of 16 blog articles that provided detailed reports of maternal deaths came from one blogging site in Nigeria alone, Linda Ikeji’s Blog.” Since findings from the blogging site are based solely on Nigeria, it would be better to limit the analysis to Nigeria instead of attempting a cross-country comparison.

***Response****:* Your statement “Since findings from the blogging site are based solely on Nigeria…” is not accurate. Not all the blogging sites were from Nigeria, as you will notice from the statement you cited from our paper above. Also see S1 File. As you will note from our objectives, we did not set out to focus solely on Nigeria, as such limiting analysis to Nigeria where there are blogs from other countries will be improper. Where possible, we have nuanced our interpretation with cross country comparisons.

Conclusion:

“When it comes to issues contributing to maternal health …. within their communities.” Authors should have discussed the role of the media in the background section instead of addressing it here. Since the authors focused on newspapers, blogs, and reports, they need to explain how these three forms of print media can help reduce maternal deaths and what policies different stakeholders can adopt using these sources.

***Response****:* We already described the role of the media in the background - a description we have further expanded following one of your comments above. In the introduction we write that, “*The media, encompassing both traditional (e.g. newspaper, television, and radio) and new/digital media (e.g. online newspaper, blogs, online reports etc.), is another source of information on health topics, including maternal deaths [10,11]. The media plays a crucial role in shaping the public’s understanding and perception of public health issues, with digital media, deemed to be particularly effective in this regard [12–14]. Beyond informing the public, the media exerts significant influence on real-world events by holding power to account, amplifying marginalised voices, and guiding the decisions of policymakers. In the area of maternal health, the media has been used to convey the severity of the maternal mortality crisis, impact of implemented interventions, and the need for improved health policies and legislation [15]”* (Page 1, Paragraph 3).

Please note that we have revamped our conclusion completely following feedback from another reviewer. We have flagged potential contribution of the media in informing the public and policy decision-making.

References:

Authors need to format article numbers and references differently. Otherwise, they may appear similar and confuse the audience.

***Response****:* Thank you for the comment. They are formatted differently. We also removed the %, where possible.

**Reviewer #3**

General Comments

Thank you for the opportunity to review this manuscript. The topic is timely and of clear relevance, particularly given the role of media in shaping public discourse and, to some extent, political and economic decision-making. Examining media representations of maternal mortality in West Africa is therefore an important and worthwhile endeavour.

***Response****:* Thank you for recognising the relevance of our study. We could not agree less with you that this is a timely topic now especially in an era of disinformation. We are immensely grateful for your very helpful feedback.

Before addressing the manuscript section by section, I wish to raise an issue related to researcher positionality. The lead author appears to be based outside the region, while the co-authors’ affiliations suggest regional representation. Given the importance of contextual understanding in media and situational analysis—especially when interpreting meanings attributed to text and images—this positionality warrants explicit acknowledgement. Clarifying how contextual expertise and local perspectives informed the analysis would strengthen the manuscript. It is also unclear whether this work originated as a graduate research project developed under the supervision of the co-authors; if so, this could be transparently stated.

***Response****:* Thank you very raising this very important point. Indeed, this was important work done as part of an MSc project at the London School of Hygiene & Tropical Medicine by the first author (KR). The student got expert support from the senior author (AB-T), who was their supervisor for the project and who is an expert in maternal and newborn health research Across Africa and in particular Anglophone West Africa where he has led or been involved in projects across all five included countries. The first author also had the support of a health media communication expert (NB) who also consolidated the contextual expertise and local perspectives that informed the analysis. In line with the journal policy, these details have now been included as part of acknowledgement (Page 18, Paragraph 2).

The introduction is presented rather lightly and would benefit from greater engagement with the broader literature on media consumption, representation, and framing, particularly in relation to maternal mortality. There are few references that reflect the historical and conceptual complexity of this field. For example, the manuscript would benefit from situating the study within existing research and investment in maternal mortality research and advocacy (MMRA) in the region.

***Response****:* Thank you to for the comment. We have reviewed the introduction to make sure it reflects more from the broader literature on the role of the media and maternal mortality, citing another review from the literature. We have written that, *“The media plays a crucial role in shaping the public’s understanding and perception of public health issues, with digital media, deemed to be particularly effective in this regard [12–14]. Beyond informing the public, the media exerts significant influence on real-world events by holding power to account, amplifying marginalised voices, and guiding the decisions of policymakers. In the area of maternal health, the media has been used to convey the severity of the maternal mortality crisis, impact of implemented interventions, and the need for improved health policies and legislation [15]”* (Page 1, Paragraph 3).

The rationale for selecting the specific countries and sub-region is not clearly articulated. While the introduction emphasises high maternal mortality rates in the countries of focus, it remains unclear whether this was the primary selection criterion, or whether practical considerations—such as the availability of English-language media sources—also influenced the choice. As currently presented, the reader is left to infer this rationale. Additionally, the opening statements are broad and predominantly negatively framed, despite the highly nuanced and heterogeneous nature of maternal mortality trends at regional and sub-regional levels. This framing relies on very limited citation and would benefit from a more balanced and contextualised discussion.

***Response****:* Thank you to for the comment. We have revised our rationale for country selection, writing, *“Because language differentially shapes how public health issues are communicated in the media [16,17], any assessment of the media’s potential utility for this purpose must be conducted within a specific linguistic context”* (Page 1, Paragraph 4)*.* The citation included is the most recent reference from the WHO. See reference: WHO, UNICEF, UNFPA, World Bank Group, UNDESA/Population Division. Trends in maternal mortality 2000 to 2023: Estimates by WHO, UNICEF, UNFPA, World Bank Group and UNDESA/Population Division. New York; 2025. Available from: <https://data.unicef.org/wp-content/uploads/2025/04/Trends-in-maternal-mortality-2020-2023.pdf>. We capture more on the heterogenous nature of maternal mortality trends in the Methods under Setting.

As a general point, I recommend a careful review of the language used throughout the manuscript. At times, the tone is overly casual for an academic article. For example, terms such as “blame culture” and phrases such as “beyond counting the numbers and statistics” are used without sufficient conceptual grounding or citation. Several sentences would benefit from revision to improve sensitivity and clarity, particularly those on page 1, lines 35–39.

***Response****:* Thank you to for your comment. It is not quite what you refer to as “overly casual”, “blame culture” is a widely recognised phenomenon in social autopsy and MPDSR literature. See ref of an article that used the same language. Kinney M V., Day LT, Palestra F, Biswas A, Jackson D, Roos N, et al. Overcoming blame culture: key strategies to catalyse maternal and perinatal death surveillance and response. BJOG. 2022;129(6):839–44. Available from: /doi/pdf/10.1111/1471-0528.16989

Following your comment, we have reviewed the entire manuscript to improve language where it was deemed necessary. We also used Grammarly to check the language.

Methods

The methods section is generally well presented. However, the content analysis procedure referenced would benefit from a clearer description, even if brief. Specifically, it would be helpful to outline how this protocol was developed, what theoretical or methodological framework underpins it, and how it was applied in the context of this study.

***Response****:* Thank you for the comment. The conceptual frameworks used in this study were already highlighted. We used *“the three-delay framework to map and assess what about maternal deaths”* (Page 4, Paragraph 5) and “*professional standards of reports as established in the Association of Health Care Journalists (AHCJ)’s principles of health reporting [24,25] and the Impress Standards Code [26] to assess how maternal deaths are reported”* (Page 4, Paragraph 6). We have further expanded the narrative under the methods to better characterise the process implemented for this Research (See Methods).

Clarification is also needed regarding the search strategy. Were “pregnant woman” and “maternal mortality” the only search terms used? If so, a justification should be provided, along with a discussion of potential limitations. Additionally, the PRISMA diagram should be explicitly referred to as Figure 1 within the text to guide the reader.

***Response****:* We have expanded our description of the search terms and how they were used (Page 3, Paragraph 3). The PRISMA is explicitly referred to as Figure 1.

**Results**

There appears to be considerable repetition between the tables and the narrative text, particularly in the presentation of qualitative findings. I recommend revising this section to reduce redundancy and instead draw the reader’s attention to the key nuances, interpretations, and implications of the quoted material—both individually and collectively.

***Response****:* Thank you for this comment. We have revised the section to reduce redundancy, as advised. We have removed some sub-headings leaving the section easier to follow with three main sub-headings (1. Characteristics of included articles, 2. What about maternal deaths are being reported? and 3. How are maternal deaths being reported?). The results have been restructured by country with nuances highlighted to make reading easier. We have removed almost all quotes in the narrative of the phase delays to reduce redundancy. The quotes left within the narrative are deemed crucial for context.

It is also unclear whether both print and electronic media sources were accessed for data extraction; this should be explicitly stated. The presentation of results using extensive numerical references following individual statements is confusing and detracts from readability, particularly in the quantitative content analysis section. While I appreciate the need to reference source articles, the current approach makes it difficult for the reader to follow the main findings. The authors may wish to consider alternative ways of presenting these references (e.g. grouped citations or table-based cross-referencing).

***Response****:* Thank you for this important comment. Only electronic i.e. digital media was accessed for this study. We have removed the word “print”, since we did not retrieve printed newspapers for the work and we focused on those newspapers which were digitally accessible via the Internet. We agree with you that the need for referencing is crucial for transparency and scientific rigour. It is not on usual that reviews such as this have many citations within the body. We already have them as grouped citations where many citations are aggregated. See example of published reviews in the journal with similar structuring. As per the journal style, the text is in black font while the citations are in red font (For example, “mothers are dying [24, 25, 78]”). This will minimise risk of confusion as has been the case in similar published papers in the journal. We have also removed % from text, where possible.

Jaguga F, Kiburi SK, Temet E, Aalsma MC, Ott MA, Maina RW, et al. A scoping review of substance use brief interventions in Africa. PLOS Global Public Health. 2024;4(10):e0003340.

Available from: <https://journals.plos.org/globalpublichealth/article?id=10.1371/journal.pgph.0003340>

Finally, the limitations section notes the use of social media to identify relevant articles on maternal mortality. However, no corresponding methodological detail is provided earlier in the manuscript. Please clarify whether social media platforms were used as a source of data, what methods were employed, and what this process yielded.

***Response****:* We appreciate you seeking clarification on this point. Please note that the social media campaign launched on this project was described in Methods already. See S2 File showing the flyer used for the campaign.

Finally, the recommendations made towards the improvement of maternal health services, including structural and economic determinants are not well placed within the article as it does not sufficiently address these factors (as per the aims). The recommendations to improve journalism are also not necessary unless there was sufficient focus on the media sources used (Blog versus mainstream digital media) and therefore it is hard to make comment on practice. It would be more useful to clear reiterate the key findings as they were identified (as per the aim/s of the study) and question the role of media, the responsibilities of the narrator and journalist given the current democratization and increasing distrust of media as a source of 'truth". These latter points are just to consider in future revision.

***Response****:* Thank you for this comment. Our objective was to understand what about and how are maternal deaths reported. We have reviewed our recommendations again. Please note that the delays identified from the review of articles highlights individual and health system factors contributing to maternal deaths. As such, this means we should discuss these findings. Similarly, implications for media practice are very much at the core of the research aim. As this was not a paper on disinformation, we do not discuss media as a source of “truth” but have flagged its importance under implications following from your comment (Page 18, Paragraph 2).

**Reviewer 1 (from Attachment)**

Thank you for the opportunity to review this important and thoughtful contribution to the field of maternal health and public health reporting. I appreciate the substantial work that has gone into this manuscript and commend the authors for addressing a critical issue in the West African context. It has been a privilege to engage with your research. Below are my detailed comments:

***Response****:* Thank you for your detailed review of our paper and indeed your kind words. Much appreciated. We are pleased to read that you agree that this review is an important contribution to the field of maternal health and public health reporting.

Introduction

1. Paragraph 4, Lines 35–42

The rationale for selecting Anglophone countries requires strengthening. The manuscript currently states only that “there appears to be no study”. The authors should explain how Anglophone media systems differ from Francophone ones, and why these differences are important for understanding maternal health reporting.

***Response****:* Thank you to for the important comment. It is a point that was also made by another reviewer. We have revised our rationale for country selection, writing, *“Because language differentially shapes how public health issues are communicated in the media [16,17], any assessment of the media’s potential utility for this purpose must be conducted within a specific linguistic context. With approximately four of five maternal deaths in West Africa occurring in the region’s Anglophone countries [1]…]”* (Page 1, Paragraph 4)*.*

2. Paragraph 4, Lines 39–41

The statement that “this analysis will reveal how expectant mothers perceive pregnancy risks” is overstated. Media analysis cannot directly measure women’s perceptions. I would strongly recommend reframing this claim to avoid over-interpretation.

***Response****:* Thank you for pointing this out. We agree with you and have now removed the statement altogether from the manuscript (Page 1, Paragraph 4)*.*

Methods

3. Lines 46–48

Although the methods section is generally detailed, some methodological gaps remain. The authors cite Macnamara (2005) but do not describe which components of the Macnamara framework were applied. A stepwise explanation of how this approach informed data extraction, coding, and analysis should be added.

***Response****:* We note this comment. Please note that Macnamara (2005) is a guidance; not a framework to be used for analysis. As stated in our paper, we closely followed its guidance on media content analysis. See reference:

Macnamara J. Media content analysis: Its uses; benefits and best practice methodology. Asia Pacific Public Relations Journal. 2005;6(1):1–34.

4. News Source Selection

The justification for selecting the specific newspapers and blogs is insufficient. No readership, circulation, or web-traffic data are provided. Please provide objective metrics (e.g., circulation figures, page-view statistics) and explain why other possible sources were excluded.

**Response**: Thank you for this comment. We have described how circulation numbers were determined by querying ChatGPT and included a supplementary file with its output for reference. The text now reads, *“To select online newspapers, four to five of the leading online newspapers in each country were selected based on their circulation numbers, which can indicate popularity. The circulation numbers were retrieved by querying ChatGPT (OpenAI, San Francisco, California, USA). The output generated was validated by asking colleagues who live in the countries if they agreed that the identified papers were the most widely read in each country. In the case that colleagues local to the country disagree with the generated list, proposition from local colleagues was prioritised”* (Page 3, Paragraph 1)*.*

5. Search Strategy (Lines 86–100)

The search terms used (pregnant woman, maternal mortality) are very narrow and risk missing relevant articles (e.g., expectant mother, woman dies in childbirth). The authors should justify the choice of terms or expand them. Additionally, please clarify why Boolean operators were not employed to enhance the search strategy.

***Response****:* Thank you for this feedback. We had only previously stated the search terms that helped us retrieve the relevant articles. As stated, we pilot tested the search strategy before full deployment and it generated the full spectrum of media reports. This was supplemented by a wide social media campaign to secure additional sources. To address your comment and in the interest of full transparency, we have now detailed all search terms tested and the final ones used to retrieve articles. From our experience in conducting this study, these terms were the most sensitive ones. The text here now reads, *“An initial preliminary search was conducted through an iterative process to pilot test several search terms (“maternal mortality”, “maternal death”, “dead woman”, “pregnant woman”, “pregnancy-related death”, “pregnancy-associated death”, “pregnancy complication” and “obstetric mortality”) and assess relevance of articles retrieved to the study. The preliminary search was conducted using the search tab function of the websites of different news source. Following this, the search terms “pregnant woman”, “maternal mortality”, and “maternal death” were assessed as most sensitive to identify relevant articles, with the presence of one or the other (“pregnant woman” OR “maternal mortality” OR “maternal death”) warranting an assessment of the identified article”* (Page 3, Paragraph 3).

6. Eligibility Criteria (Lines 101–111)

Requiring articles to include the woman’s name and contain more than 250 words may introduce selection bias and exclude culturally sensitive reporting. The authors should justify the 250-word threshold with evidence from the literature and discuss the ethical implications of requiring the deceased woman’s name.

***Response****:* Thank you for this comment. It is not quite clear how the name could have introduced bias but we understand your concern with the word count threshold. For the name, we needed this information in the report to ensure that the veracity of the reporting was not in doubt. Simply saying, “a woman” or “Woman X” died could be a fictitious story, especially with blogs. However, a name added lends some credibility to the report. You will see in our PRISMA diagram that we flagged that we excluded one article because we had “concern on authenticity of report (n = 1)”. For the word count threshold, as mentioned, we pilot-tested this approach before applying it. Articles <250 words did not have sufficient textual richness to allow us fully to characterise and understand the obstetric cause of death. We have now explicitly stated this as a justification for taking this approach now, writing that *“Our preliminary screening of all retrieved articles revealed that articles with more than 250 words tended to have sufficient textual depth needed for meaningful content analysis, including the identification of themes, frames, and discursive patterns [19]. On the other hand, shorter reports typically lacked the narrative and contextual complexity required for comprehensive understanding of the cause of the maternal death”* (Page 3, Paragraph 4). To ensure we fully address your concern, we have flagged this as a potential limitation while highlighting the reason for setting the benchmark and stating we do not believe it would have altered our findings. We have done this by adding that “*Fifth, we included articles with 250 words or more. While this approach might have introduced some bias into our findings, our review of the less detailed articles presented in S1 File suggests that, although their inclusion would have increased the number of eligible articles, the overall conclusions would not have changed”* (Page 16, Paragraph 2). You will see in S1 File, we report the number of these undetailed reports (23) including 12 from Nigeria and none from The Gambia.

7. Data Extraction and Reliability

Although the use of Google Sheets and NVivo is appropriate, the manuscript does not describe the number of coders, their training, or intercoder reliability. Furthermore, no reliability statistics (e.g., Cohen’s kappa) are reported despite both quantitative and qualitative coding. The authors should report reliability measures or explain how coding disagreements were resolved.

***Response****:* We are immensely grateful for your comment. We have now stated clearly that, *“Two coders (KR and AB-T) independently coded all selected articles using the coding sheet. As per best practice, inter-coder reliability for the quantitative content analysis was assessed for agreement between coders using Cohen's kappa (κ) [19]. Following an iterative process, we achieved an inter-coder reliability (κ) of 0.92 [S2 File], with κ >0.75 accepted as excellent [28]*” (Page 5, Paragraph 2). *We have also included a supplementary file detailing our calculation of κ.* Please note that for the qualitative component, we deployed a mostly inductive coding. While intercoder reliability ensures multiple coders agree on data interpretation, it is generally considered unnecessary or inappropriate in purely interpretive or inductive studies that embrace subjective, multiple realities. Researchers argue that calculating numerical reliability (e.g., Cohen's Kappa) imposes quantitative, positivist, or "single truth" standards on inherently subjective, interpretive data. In lieu, we implemented a detailed audit trails and debriefing meetings. This ensured that we were able to represent the multiple realities of the text from the lens of the two coders. It is their blended understanding of what has been reported in the media that has been presented as findings. We have explained this in the manuscript and included relevant references to justify our approach.

Results

8. The results section is highly descriptive. The authors are encouraged to present the findings more concisely. Consider separating the results into descriptive characteristics and patterns of reporting to improve structure.

***Response****:* Thank you for your comment. We already separated the section along your suggested lines. To ensure this is clear, the section now has three main sub-headings (1. Characteristics of included articles, 2. What about maternal deaths are being reported? and 3. How are maternal deaths being reported?).

9. Qualitative Findings (Lines 225–293)

The qualitative synthesis reads more like a summary of article content than a thematic analysis. The authors should organise the findings around explicit themes (e.g., financial barriers, provider negligence, delays in obtaining care) and present them systematically.

***Response****:* We have not used thematic analysis but rather framework analysis. We have ensured more cautious framing across the entire discussion.

Discussion

10. Paragraph 2, Lines 420–434

The interpretation is generally strong; however, some statements are speculative, such as attributing the rise of blogs to increased pandemic-related readership, without supporting evidence. These claims should be substantiated with references or revised for caution.

***Response****:* Thank you for your comment. We have ensured more cautious framing across the entire discussion and ensured that claims are substantiated with references or revised for caution.

11. Paragraphs 3–5, Lines 435–479

Parts of the discussion read as advocacy rather than scientific analysis. To maintain academic rigor, the authors should minimise editorial tone and ensure that interpretations remain firmly grounded in the data.

***Response****:* We have revised the entire discussion to make sure it remains firmly grounded in the data.
