# Supplementary material for: Reporting maternal deaths in Anglophone West Africa: A media content analysis of articles published online between 2015 and 2025
Source: PLOS Glob Public Health. 2026 Apr 6;6(4):e0006254. doi: 10.1371/journal.pgph.0006254 (PMC13052848; doi:10.1371/journal.pgph.0006254)
Supplement: Attachment — Submitted filename: Response to Peer review comments_final_v2.docx [file pgph.0006254.s010.docx]

11^th^ March 2026

Prof Catherine Kyobutungi

Prof Manuela de Allegri

Editors-in-Chief

Dr Julia Robinson

Executive Editor

Dr Emmanuel Olamijuwon

Academic Editor

PLOS Global Public Health

Dear Editors,

**Re: Reporting maternal deaths in Anglophone West Africa: A media content analysis of articles published online between 2015 and 2025**

We appreciate the opportunity to revise and resubmit our paper, and the comments provided by the editor and reviewers, which have enabled us to strengthen the manuscript.

We have carefully considered all comments and have addressed each point comprehensively. Below, we provide a point-by-point response to the reviewers' feedback, detailing the revisions made to the manuscript.

We took the opportunity to update the review to include newer articles that have emerged in the media since our first submission, updated the abstract based on new changes made, created new supplementary files, and reviewed language in the entire manuscript to ensure clarity. We also made a change to the title to make it more reflective of the content of the paper.

We believe these revisions have strengthened the methodological rigour, theoretical grounding, and clarity of our study, and we hope the manuscript is now suitable for publication in PLOS Global Public Health.

Thank you for your consideration.

Kind regards,

Aduragbemi Banke-Thomas

(On behalf of all authors)

**Reviewer #1:**

The authors have made substantial revisions in response to the reviewer comments. The methodological procedures are now clearer, particularly with the inclusion of intercoder reliability and improved explanation of the analytic framework. The Results and Discussion sections are better organised and more cautiously interpreted, which strengthens the overall rigour of the manuscript. The rationale for focusing on Anglophone countries could still be strengthened by briefly clarifying how differences between Anglophone and Francophone media systems may shape patterns of maternal death reporting. Nonetheless, the authors have addressed the major concerns satisfactorily, and I recommend the manuscript for acceptance.

***Response****:* Thank you for your very helpful and thorough review of our paper. We are glad that you now view the methodological procedures are now clearer, the Results and Discussion sections better organised and more cautiously interpreted. Thank you for recommending the paper for acceptance. We have taken up your challenge to further strengthen the rationale. It now reads, “Data from the media offers a viable alternative, particularly since it imposes no direct cost on the government. Yet, to date, no study reviewing media content on maternal deaths in West Africa has been published to date. Analysis attempting to address this knowledge gap must control for reporting language, recognising that distinct journalistic traditions and media systems are inextricably linked to linguistic heritage and context [16,17]. With recognition that language differentially shapes how public health issues are communicated in the media [18] and approximately four of five maternal deaths in West Africa occurring in the region’s Anglophone countries [1], our study aimed to conduct a media content analysis to examine what about and how maternal deaths are being reported in digital media in Anglophone West Africa”.

Two new key references have been added to buttress the point:

16. Cotter C. Diversity awareness and the role of language in cultural representations in news stories. J Pragmat. 2011;43(7):1890–9.

17. Bonin G, Dingerkus F, Dubied A, Mertens S, Rollwagen H, Sacco V, et al. Quelle Différence?: Language, culture and nationality as influences on francophone journalists’ identity. Journal Stud. 2017;18(5):536–54.

In addition to the two we had used previously:

1. WHO, UNICEF, UNFPA, World Bank Group, UNDESA/Population Division. Trends in maternal mortality 2000 to 2023: Estimates by WHO, UNICEF, UNFPA, World Bank Group and UNDESA/Population Division [Internet]. New York; 2025. Report. Available from: https://data.unicef.org/wp-content/uploads/2025/04/Trends-in-maternal-mortality-2020-2023.pdf

18. Georgiou GP. Words are not just words: how the use of media language in the COVID-19 era affects public health. Epidemiol Health. 2021;43:e2021072.

We have also added a sentence to the limitations writing, “By restricting our analysis to Anglophone countries, we have endeavoured to hold the overarching media system and journalistic traditions influenced by language relatively constant, allowing for a more precise and undistorted analysis of how localised sociocultural, institutional, and editorial factors specifically influence the framing and reporting of maternal deaths”.

**Reviewer #3**

The manuscript has been revised and is improved from the first version - all reviewer comments are very well addressed. I do still see scope for additional revision to further improve the grammar and sentence construction before it is ready for publication.

***Response****:* Thank you for your comment. We certainly agree that there was a need to have a thorough review of the paper one more time. We have done an end-to-end review to pick up any issues with grammar and sentence construction. Please see the multiple tweaks made in the updated manuscript.
