# Supplementary material for: Reporting maternal deaths in Anglophone West Africa: A media content analysis of articles published online between 2015 and 2025
Source: PLOS Glob Public Health. 2026 Apr 6;6(4):e0006254. doi: 10.1371/journal.pgph.0006254 (PMC13052848; doi:10.1371/journal.pgph.0006254)
Supplement: Attachment — Submitted filename: Reviewer comments.docx [file pgph.0006254.s008.docx]

Thank you for the opportunity to review this interesting piece of work. The authors have made substantial revisions in response to the reviewer comments. The methodological procedures are now clearer, particularly with the inclusion of intercoder reliability and improved explanation of the analytic framework. The Results and Discussion sections are better organised and more cautiously interpreted, which strengthens the overall rigour of the manuscript. The rationale for focusing on Anglophone countries could still be strengthened by briefly clarifying how differences between Anglophone and Francophone media systems may shape patterns of maternal death reporting. Nonetheless, the authors have addressed the major concerns satisfactorily, and I recommend the manuscript for acceptance.
